# The Debate about Dendritic Cells and Macrophages in the Kidney

**DOI:** 10.3389/fimmu.2015.00435

**Published:** 2015-09-01

**Authors:** Catherine Gottschalk, Christian Kurts

**Affiliations:** ^1^Institute of Experimental Immunology, Rheinische Friedrich-Wilhelms-Universität Bonn, Bonn, Germany

**Keywords:** dendritic cells, macrophages, kidney, flow-cytometry, glomerulonephritis

## Abstract

The mononuclear phagocyte system includes macrophages and dendritic cells (DCs), which are usually classified by morphology, phenotypical characteristics, and function. In the last decades, large research communities have gathered substantial knowledge on the roles of these cells in immune homeostasis and anti-infectious defense. However, these communities developed to a degree independent from each other, so that the nomenclature and functions of the numerous DC and macrophage subsets overlap, resulting in the present intense debate about the correct nomenclature. This controversy has also reached the field of experimental nephrology. At present, no mutually accepted way to distinguish renal DC and macrophages is available, so that many important roles in acute and chronic kidney disease have been ascribed to both DCs and macrophages. In this perspective article, we discuss the causes and consequences of the overlapping DC–macrophage classification systems, functional roles of DCs and macrophages, and the transferability of recent findings from other disciplines to the renal mononuclear phagocyte system from the nephrologist’s point of view.

## Introduction

The current intense debate regarding the classification and nomenclature of dendritic cells (DCs) and macrophages has reached also the field of experimental nephrology. Numerous kidney diseases are immune mediated, such as the different forms of glomerulonephritis, and research over the last years has described important, yet overlapping roles of both cells types.

Macrophages and DCs are often considered distinct cell types based on their morphology and function. Macrophages were defined as large vacuolar cells that are highly phagocytic and modulate immune responses by production of immune mediators (1, 2), whereas DCs were characterized as stellate migratory cells that act as sentinels in non-lymphoid tissues and enter lymphoid tissues upon antigen encounter, present antigen and subsequently activate naïve T lymphocytes (3–5). Following these original descriptions, two research areas developed that more or less independently studied macrophages and DCs. This artificial separation has contributed to the emergence of different names for similar or the same cell types, thereby adding to the current confusion about their identity and function. In particular, advances in multi-color flow cytometry and gene-analyses enabled researchers to define many DC and macrophage subsets by the expression of a variety of surface molecules (6). As cell surface markers are easy to determine, they are widely used to classify mononuclear phagocytes, although they are rather unspecific and their expression patterns in the murine and human systems differ substantially.

Also in the kidney, surface markers and functional parameters have been used to propose several classification systems of mononuclear phagocytes. However, these systems overlap, comparable to the situation in other non-lymphoid organs, resulting in great uncertainty among experimental nephrologists regarding the correct terminology. Here, we discuss the present state of our knowledge on renal mononuclear phagocytes in health and disease and problems resulting from the current nomenclature debate from a nephrologist point of view.

## The Network of Renal DCs and Macrophages

The kidney parenchyma consists of the outer renal cortex and the inner renal medulla. Numerous individual functional units, the nephrons, span both compartments. The cortex contains glomeruli and proximal tubuli of the nephrons, which generate the primary urine. The medulla contains the loop of Henle, which generates a high osmolarity that is required for water reabsorption from the primary urine. The distal tubules end in collecting ducts through which the concentrated urine is transported into the renal pelvis and on through the ureters into the bladder. The space between the tubules is known as tubulointerstitium and contains blood vessels, fibroblasts, and numerous cells of the hematopoietic system that had been classified by pathologists as constituents of the reticuloendothelial system [reviewed in Ref. (7)].

Early immunological studies had classified the tubulointerstitial mononuclear cells as macrophages due to their F4/80 expression (8). During the early 1990s, several groups reported that these tubulointerstitial cells morphologically resembled DCs in humans and rodents (9–12), whereas cells with the typical morphology of macrophages were described to reside mainly in the kidney capsule, the intravasal lumina, and the pelvic wall of healthy kidneys (13). The use of CX_3_CR1-reporter mice and live cell imaging illustrated the intricate tubulointerstitial network of dendritiform processes that these cells use to constantly probe the environment, suggestive of DCs in action (14–16). The nomenclature debate intensified when it became clear that the vast majority of renal mononuclear phagocytes possess the phenotype CD11c^+^ CD11b^+^ F4/80^+^ CX_3_CR1^+^ (17), which allows classification of both macrophages and DCs.

Notably, CX_3_CR1 exhibits relative organ specificity for renal mononuclear phagocytes: these cells were >50% reduced in the kidneys, but not in other organs (except the intestine) of CX_3_CR1-deficient mice. This may be explained by the comparatively high renal expression of its ligand CX_3_CL1 (18). Notably, those CX_3_CR1^+^ phagocytes that co-express CD11c and exert DC functionality were reduced even by more than 75% (18–20). This may result from an effect of CX_3_CR1 on CD11c expression, but this has yet to be shown. Interestingly, CX_3_CR1 regulated the numbers of the CD11c^+^ and the CD11c^−^ renal mononuclear phagocytes by different mechanisms: it promoted homeostatic and inflammatory recruitment of the former, whereas it prevented *in situ* proliferation of the latter under inflammatory conditions (20). Assuming that CD11c distinguishes renal DCs and macrophages, this difference would be consistent with recent reports that the number of tissue macrophages is regulated by local proliferation (21), whereas DC numbers are usually thought to be regulated by immigration and emigration (22).

The kidney also contains a minor subset of CD103^+^ DCs, which constitute <5% of all renal CD11c^+^ phagocytes and lack expression of CX_3_CR1, CD11b, and F4/80 (23), whose function currently is unclear. There are neither CD11b^+^ CD103^+^ DCs nor plasmacytoid DCs in the healthy kidney (24).

## Functionality and Phenotype of Renal Mononuclear Phagocytes

Researchers from both, the DC and the macrophage fields, have investigated kidney mononuclear phagocytes defined by cell surface markers in homeostasis and models of renal disease. Many important roles were shown in models of acute renal injury and in chronic immune-mediated kidney disease (Table 1), such as cytokine production or T cell-crosstalk in response to tissue injury or infection (17, 25–33). However, none of these functions is generally accepted to be exclusive for DCs or macrophages. Moreover, many nephrologists trained by the DC and macrophage communities still use CD11c and F4/80 to identify DCs and macrophages, respectively (see Table 1), even though 70–90% of renal mononuclear phagocytes co-express these two markers (17), implying that they studied cellular subsets that largely overlap. Also, the tools used for loss-of-function studies cannot clearly discriminate between DCs and macrophages: CD11c–DTR mice are used to deplete kidney DCs, CD11b–DTR mice for depleting kidney macrophages but the expression of CD11c and CD11b on kidney mononuclear phagocytes is too heterogeneous for this black-and-white thinking (34). Clodronate liposomes are used for both purposes (35–38). All kidney mononuclear phagocytes are phagocytic (34) which might render them sensitive to clodronate liposomes.

**Table 1:** **Summary of the functions of mononuclear phagocyte subsets in renal diseases, which have been attributed to either renal DC or macrophages, based on marker expression and/or disease attenuation or aggravation after cell depletion**.

Disease	Function and associated cell type	Classification of associated cell types
**Acute renal injury**	**Pro-inflammatory**	**Pro-inflammatory**
Ischemia/re-perfusion	I. TNFa secretion *DC* (26, 63, 64) *Macrophages* (63, 64) II. Th activation *Macrophages* (63)	*DC* CD45^+^, CD11c^+^, MHCII^+^, CD11b^+^, CD16^+^, F4/80^+^, CD68^+^, CD4^−^, CD8^−^, CD205^−^, 33D1^−^, CD169^−^ CD204^−^ (26) *Macrophages* Sensitive to liposomal dichloromethylene bisphosphonate (clodronate liposome) treatment, F4/80^+^ (63) Sensitive to clodronate liposome treatment (64)
	**Anti-inflammatory**	**Anti-inflammatory**
	I. Tissue regeneration *DC* (33) *Macrophages* (67) II. Suppression of TNFa, IL-6, CXCL2, CCL2 production by IRF4 upregulation *DC* (65) III. Prevention of renal failure *DC* (66)	*DC* Sensitive to clodronate liposome treatment, CD45^+^, MHCII^+^, CD11c^+^, F4/80^+^ (33) Sensitive to clodronate liposome treatment, CD45^+^, MHCII^+^, CD11c^+^ (65) Sensitive to clodronate liposome treatment, CD11b^+^ (66) *Macrophages* Sensitive to clodronate liposome treatment, F4/80^+^ (67)
Unilateral ureter obstruction (UUO)	**Pro-inflammatory**	**Pro-inflammatory**
	I. Antigen presentation to CD4^+^ T cells *DC* (27) II. Accumulation of Th17 cells *DC* (28) III. TNFa, TGFb production *DC* (28, 68) *Macrophages* (68) IV. Tubular apoptosis *DC* (68) *Macrophages* (68) V. Renal fibrosis *DC* (68, 69) *Macrophages* (68, 69)	*DC* CD11c^+^, T cell stimulatory, phagocytotic (27) CD45^+^, CD11c^+^, F4/80^+^, Ly6C^−^ or CD45^+^, CD11c^+^, F4/80^−^, Ly6C^−^, sensitive to clodronate liposome treatment (28) CD45^+^ CD11c^+^, F4/80^+^ (sensitive to clodronate liposomes) or F4/80^−^ (not sensitive to clodronate liposomes) (68) *Macrophages* CD45^+^ F4/80^+^, CD11c^−^, sensitive to clodronate liposomes (68) CD45^+^, CD11b^+^, Csfr1R-GFP^+^, CD11c^−^; depletion in CD11b–DTR mice (69)
Adriamycin nephropathy, cisplatin nephropathy, crystal nephropathy	**Pro-inflammatory**	**Pro-inflammatory**
	I. Aggravation of kidney injury in adriamycin-induced nephropathy *Macrophages* (25) II. IL-1b secretion after inflammasome activation *DC* (29)	*DC* *In vitro* studies with bone marrow derived DC; renal CD45^+^, CD11c^+^ cells; sensitive to clodronate liposome depletion and diphtheria toxin in CD11c–DTRg mice (29) *Macrophages* CD45^+^, MHCII^+^, CD11c^+^, F4/80^+^, CD68^+^, CD204^+^, CD206^+^, CD103^−^; morphology, phagocytic capacity, ontogeny (25)
	**Anti-inflammatory**	**Anti-inflammatory**
	I. Protective against cisplatin nephropathy, induction of IL-10 *DC* (70)	*DC* CD45^+^, MHCII^+^, CD11c^+^, CD11b^+^, F4/80^+^; morphology of GFP^+^ cells in CD11c–DTRtg mice (70)
**Chronic renal disease**	**Accumulating**	**Accumulating**
Glomerulonephritides	I. Population changes during nephrotoxic nephritis *DC* (17)	*DC* CD11c^+^, CD11b^+^, F4/80^+^; morphology, lysosomal content, phagocytic activity, microbicidal effector functions, expression of T cell costimulatory molecules, T cell activation (17)
	**Pro-inflammatory**	**Pro-inflammatory**
	I. Crescent formation *Macrophages* (71) II. T cell infiltration and activation *DC* (32, 72) III. Chemokine expression *DC* (73)	*DC* MHCII^+^, CD11c^+^, F4/80^−^ (72) MHCII^+^, CD11c^+^ CD11b^+^, CD8^−^, B220^−^; depletion in CD11c–DTR mice; antigen presentation and T cell activation function (32) Chemokine expression by CD11b^+^ CD11c^+^ DC was analyzed in lymphoid organs (73) *Macrophages* Sensitive to diphtheria toxin in CD11b-DTR mice, CD68^+^ (71)
	**Anti-inflammatory**	**Anti-inflammatory**
	I. Induction of IL-10 secretion by CD4 T cells *DC* (31) II. Recruitment of regulatory CXCR6^+^ iNKT cells *DC* (74)	*DC* Morphology; MHCII^+^, CD11c^+^, CD11b^+^, sensitive to diphtheria toxin in CD11c-DTR mice (31) CD45^+^, CD11c^+^, depletion in CD11c-luciDTR mice (74)
**Infection**	**Anti-infectious**	**Anti-infectious**
	I. Bacterial clearance *DC* (18, 30) II. Candida protection *Macrophages* (19) III. Response to infectious stimuli, chemokine secretion, migration *DC* (75)	*DC* MHCII^+^, CD45^+^, CD11c^+^, CD11b^+^, F4/80^+^, CX_3_CR1^+^ CD103^−^; depletion in CD11c–DTR mice (18, 30) Enrichment by Flt3L administration, sorted by CD11c purification (75) *Macrophages* MHCII^+^, F4/80^+^, CD11b^+^, CD11c^lo^; morphology (19)

The consequence of this overlap is well illustrated by two recent studies examining how CX_3_CR1 affects renal disease: both studies agreed that mononuclear phagocytes are substantially reduced in the kidneys of CX_3_CR1-deficient mice. However, one of them noted a higher susceptibility to renal candidiasis and attributed this to the loss of renal macrophages (19), while the other documented protection against glomerulonephritis and assigned this to the loss of renal DCs (18). A possible explanation for this different classifications is that glomerulonephritis is driven mostly by phagocytes in the kidney cortex, in which glomeruli are located, whereas anti-infectious activity seem to be primarily due to phagocytes in the medulla, through which pathogens enter the kidney (18). Medullary phagocytes express significantly less CD11c than those in the cortex, which may bias their classification as DCs. The causes for these phenotypical and functional differences between medullary and cortical mononuclear phagocytes are unknown, but may result from differences in osmolarity, pH, and oxygen tension between these compartments, to which the mononuclear phagocytes may adapt. This would be in line with the current view that the tissue microenvironment dictates the organ-specific plasticity of macrophages (39, 40), and thus, perhaps also of renal mononuclear phagocytes.

## Re-Defining Kidney Mononuclear Phagocyte Nomenclature

The current definitions of renal DCs and macrophages are not mutually exclusive, so that renal mononuclear phagocytes may fulfill the definitions of both cell types simultaneously. This creates confusion, especially among those nephrologists that are more interested in disease relevance than in semantics. A recent proposal for a unified nomenclature has been based on cellular ontogeny: it proposes an initial division of mononuclear phagocytes into macrophages, monocytes and monocyte-derived cells and DCs (so-called “level 1 nomenclature”) (41). This classification was based on the following facts: (1) most adult macrophages in tissues are successors of an embryonic precursor and maintained through self-renewal (42–46), (2) a common monocyte progenitor (cMoP) exists, which gives rise to monocytes (47), and (3) conventional DC (cDC) and plasmacytoid DC but not monocytes or macrophages arise from a common DC precursor (CDP) (48, 49). Thus, tissue-resident macrophages were classified by their origin from embryonic (yolk sac and fetal monocytes)-derived erythro-myeloid progenitors (46, 50) and DC were classified as cells arising from hematopoietic stem cell-derived precursors, identified by genetic tracing via DNGR1 (CLEC9A) (51), which are distinct from monocyte/macrophage precursors. Finally, monocyte-derived cells differentiate from cMoP that can exert macrophage- or DC-like functions and express markers associated with either (41). This classification does not resolve the question whether monocyte-derived macrophages and monocyte-derived DCs are ontogenically distinct or whether one cell type displays high plasticity in different microenvironments. To include cell function, location, and morphology, the authors suggested to add a “level 2” nomenclature to the level 1 classification (41).

While this nomenclature proposal might bring order into the ever increasing numbers DC and macrophage subsets, one major concern remains: without fate mapping tools, the origin of a phagocyte in a given tissue is usually not apparent, so that surrogate markers need to be used. Several markers for distinguishing phagocytes derived from different precursors are currently being discussed, but as we shall see below, they fail to discriminate renal DCs and macrophages.

One of these markers, CD64, alone or in combination with CCR2 or MerTK, has been reported to identify monocyte-derived macrophages and to be able to discriminate DC from non-DC in the intestine, the muscle and spleen (52–55), and the skin (56). DNGR1, when combined with genetic fate mapping technology, was shown to mark CDP and pre-DC (51), whereas Csf1r can be used for fate mapping of yolk sac derived (myb independent) tissue macrophages (46). In the kidney, most mononuclear phagocytes express CD64, low levels of CD11b and high levels of F4/80, which is not the case in other organs. However, 30% of CD64^+^ cells co-expressed the DNGR1-fate mapper, indicating that CD64 expression, despite the evidence for specificity in other organs, does not differentiate CDP-derived from monocyte-derived cells in the kidney (51). Similarly, another fate mapping study that used Myb and PU.1 dependency for defining CD11b^hi^ monocytes or macrophages and F4/80^bright^ tissue macrophages derived by adult or embryonic hematopoiesis, respectively, found a dual origin in kidney macrophages as well (45). These findings highlight the difficulties when basing cellular classification solely on ontogeny when ontogeny is based on surrogate markers. Furthermore, transferring ontogeny-based nomenclature to human mononuclear phagocytes in tissue might prove impracticable.

A classification approach based on transcriptome analysis reported that CD11c^+^ MHC II^+^ cells in the kidney expressed a set of core DC markers characteristic of DCs in non-lymphoid tissues, that is absent from macrophages, including Zbtb64, Flt3, and CCR7 (57). These “core DC markers” had been defined by analyzing cDCs except the CD11b^+^ non-lymphoid tissue–DC, because of the great heterogeneity of CD11b^+^ cells. However, these constitute the vast majority of kidney mononuclear phagocytes.

Another classification approach is based on mononuclear phagocyte functionality. However, observed functions generally represent a snapshot of a cell within a specific context and time frame. Demonstrating that a phagocyte performs a given function under certain conditions at a certain time-point does not imply that this is a general feature of this cell. Furthermore, there is no clear demarcation between exclusive DC and macrophage functions. For example, macrophages phagocytose and degrade material. However, under certain conditions DCs do that too, albeit less efficiently [reviewed in Ref. (58)]. On the other hand, DCs classically activate naïve T cells, but macrophages can do that too, albeit less efficiently (59, 60). Furthermore, the ability to stimulate T cells is difficult to determine on a single cell basis. A recent study differentiated renal mononuclear phagocytes into five phenotypically and functionally distinct populations (34). In that study, mononuclear phagocyte populations were differentiated by CD11c, CD11b, F4/80, CD103, CD14, CD16, and CD64 expression in juvenile and adult mice of different strains. Functional analyses and fate mapping studies were used for further characterization. In line with the complexity of kidney mononuclear phagocyte subsets observed by others and us (17, 45, 51), the study revealed that all subsets expressed CD68 that is usually used to identify macrophages and that all subsets were phagocytic but showed differences in their antigen presentation capacity. Fate mapping experiments identified one population with a dual origin, two populations that were closely related to monocytes, whereas the remaining two were not. Notably, the largest population not only showed the phenotypical and functional characteristics of reparative macrophages (M2) but also had significant antigen presentation function and most likely emigrated from the kidney under inflammatory conditions. Additionally, this population differed significantly between mouse strains, which might explain immunological differences between those strains. The authors concluded that functions are more related to context than separate lineage and suggested their marker combination as an unbiased approach to identify kidney mononuclear phagocyte populations (34). These findings are consistent with recent concepts that macrophage fine differentiation is shaped by the tissue microenvironment (39, 40).

## Concluding Remarks

As a consequence of the separate development of the DC and macrophage research communities, the functional and phenotypic definitions of these cell types overlap substantially. Thus, scientists from both communities often study the same cells, perhaps unaware of, or ignoring progress and concepts in the other field. The false assumption that classifying a mononuclear phagocyte as a macrophage implies that it is not a DC, and vice versa, hampers communication between researchers from both fields. Some studies have focused on arguing about subsets and semantics (61), perhaps hoping to “claim territory” for their own communities. This may result in highly citable or controversial publications, but it does not advance our understanding of mononuclear phagocytes, neither in the kidney nor elsewhere.

An overlapping classification system, such as the existing one, is certainly not desirable. An improvement is needed. It is unrealistic to assume that either the DC or the macrophage community will accept the nomenclature of the other field. Drawing a line that segregates mononuclear phagocytes into DCs or macrophages will unlikely be acceptable to both fields. Furthermore, there are currently no unambiguous discriminatory parameters; for any new parameters introduced, exceptions are reported quickly, such as for CD64 and DNGR1-fate tracking in the kidney. Still, an improved classification system is needed. How can we reach a consensus?

First, the purpose of the revised classification system needs to be defined. Clinicians are interested in cellular entities that are useful for diagnostic or therapeutic purposes and translational immunologists often study the functions of cellular subsets. Basic immunologists may favor ontogeny, which is biologically the cleanest and most logical approach. However, mononuclear phagocytes adapt their gene enhancer landscape according to the tissue of residence independently of the precursor they originated from (39), an ontogeny-based nomenclature may lead to different cell types with similar functionality, or to cells of the same name with different functionality depending on the organ they reside in. Moreover, the origin of a mononuclear phagocyte in a given tissue is not obviously apparent, because unique discriminatory parameters are missing. Thus, ontogeny, although theoretically logical, will be difficult to use for routine research. At the end of the day, a classification system needs to be convenient and feasible, or it will not be used.

The late Ralph Steinman remarked “The DC is a functional state” (personal communication). Indeed, at the age of single cell transcriptomics, it becomes clear that several transcriptional programs may run simultaneously in individual mononuclear phagocytes, and confer a spectrum of functionalities that are more or less consistent with the current concepts of a DC, of a macrophage, or both. Current technical advances will undoubtedly allow distinguishing far more functional states of mononuclear phagocytes. In the field of renal immunology, experts coming from the DC and macrophage communities have jointly suggested avoiding the DC–macrophage controversy altogether by referring to mononuclear phagocytes (preferentially using a “catchier” name for these cells), with different degrees of DC- or macrophage-, or other functionalities (62). It remains to be seen whether basic immunologists and scientists studying mononuclear phagocytes in other organs feel that this is useful or not.

## Conflict of Interest Statement

The authors declare that the research was conducted in the absence of any commercial or financial relationships that could be construed as a potential conflict of interest.
